# Mycorrhizal Inoculation Enhances Drought Resilience in Citrus Seedlings of Two Cultivars by Modulating Gas Exchange and Hormonal Signaling

**DOI:** 10.3390/plants15030505

**Published:** 2026-02-06

**Authors:** Josefa María Navarro, Asunción Morte, Juan Gabriel Pérez-Pérez

**Affiliations:** 1 Equipo de Riego y Fisiología del Estrés, Instituto Murciano de Investigación y Desarrollo Agrario y Medioambiental (IMIDA), 30150 La Alberca, Murcia, Spain; 2 Departamento de Biología Vegetal (Botánica), Facultad de Biología, Campus de Espinardo, Universidad de Murcia, 30100 Espinardo, Murcia, Spain; amorte@um.es; 3 Centro para el Desarrollo de la Agricultura Sostenible, Instituto Valenciano de Investigaciones Agrarias (IVIA), 46113 Moncada, Valencia, Spain; perez_juaperb@gva.es

**Keywords:** ‘Cleopatra’ mandarin, alemow, arbuscular mycorrhizal fungi, abscisic acid, stomatal conductance, water-use efficiency, *Rhizophagus irregularis*, *Funneliformis mosseae*

## Abstract

Water scarcity and climate variability threaten citrus production in semi-arid regions, requiring strategies to improve drought resilience. This study evaluated the physiological and hormonal responses of two citrus cultivars, alemow (*Citrus macrophylla* Wester) and ‘*Cleopatra*’ *mandarin* (*Citrus reshni* Hort. Ex Tanaka), inoculated with arbuscular mycorrhizal (AM) fungi (*Rhizophagus irregularis* + *Funneliformis mosseae*) and subjected to drought stress imposed by progressive soil drying (water withholding) and quantified by volumetric soil water content (*θ_v_*) classes: >0.20 cm^3^ cm^−3^ (well-watered), 0.05–0.20 cm^3^ cm^−3^ (moderate drought), and <0.05 cm^3^ cm^−3^ (severe drought). Gas exchange, plant water status, and abscisic acid (ABA) dynamics were monitored to assess cultivar-specific effects of AM symbiosis. Under well-watered conditions, +AM plants exhibited higher photosynthetic rates than non-inoculated plants, with a stronger response in Macrophylla. During drought, contrasting patterns emerged: +AM Macrophylla maintained higher stomatal conductance and photosynthesis, with foliar ABA increasing only under severe stress, suggesting that non-hormonal mechanisms support gas exchange. In Cleopatra, AM inoculation was associated with higher root-derived ABA and earlier stomatal closure, suggesting a more conservative water-use strategy under soil drying conditions; however, the benefits were limited to moderate stress and decreased beyond a stomatal conductance threshold. These findings reveal that AM symbiosis enhances drought resilience through contrasting mechanisms: hydraulic stabilization predominates in Macrophylla, whereas hormonal (ABA-mediated) regulation drives the response in Cleopatra. This cultivar-dependent modulation highlights the importance of developing AM-based strategies adapted to each cultivar for effective citrus drought management. Combining AM inoculation with irrigation-saving practices could improve water productivity and support climate-smart citrus production.

## 1. Introduction

In recent decades, climate change has increased the frequency and intensity of drought events, posing a serious threat to fruit crop productivity and sustainability, especially in Mediterranean regions [1,2]. Citrus crops play a key role in global fruit production and are economically important in these areas. Their strong dependence on irrigation makes them particularly vulnerable to water scarcity, highlighting the need for drought-resilient strategies in semi-arid environments. In citrus, drought stress causes significant reductions in leaf water potential, stomatal conductance, and photosynthetic activity, which can negatively impact fruit set, size, and sugar content [3,4,5].

Rootstocks play a key role in determining scion vigor, water relations, and tolerance to abiotic stress, acting as physiological regulators of the plant’s response to environmental challenges; in fact, several studies have demonstrated that citrus rootstocks vary in their water-use efficiency, stomatal behavior, and hormonal regulation under drought conditions [6,7,8,9]. For example, Clemenules mandarin grafted onto different rootstocks showed contrasting responses in vegetative growth, gas exchange, and fruit quality under deficit irrigation conditions [10], while Lane Late sweet orange exhibited rootstock-dependent variation in stomatal conductance [9]. In Fino 49 lemon, water-use efficiency was improved independently of root-to-shoot ABA signaling, revealing the complexity of hormonal regulation in drought adaptation [11]. More recently, Carrizo citrange was shown to enhance the scion’s antioxidant capacity under combined drought conditions and heat stress, whereas Cleopatra mandarin failed to induce an effective antioxidant response, resulting in greater stress injury [12]. These findings reinforce the importance of rootstock selection as a key agronomic tool to improve fruit resilience under climate stress.

Arbuscular mycorrhizal (AM) fungi have also emerged as promising biotechnological allies to improve drought tolerance in fruit crops. AM symbiosis enhances water and nutrient uptake, increases root hydraulic conductivity, and modulates hormonal signaling, especially ABA, leading to improved physiological performance under stress [2,13,14,15]. AM fungi also help reduce oxidative damage by boosting antioxidant enzyme activity and modifying root fatty acid saturation, which stabilizes membranes during dehydration processes [16]. In citrus, AM inoculation has been shown to maintain higher leaf water potential, improve water-use efficiency, and reduce reactive oxygen species (ROS) accumulation compared to non-inoculated plants under drought conditions [17,18].

Although the general benefits of AM inoculation under drought conditions are well documented, few studies have explored how cultivars used as rootstocks interact with AM symbiosis to influence drought responses in citrus. Most research has focused on either cultivar performance or AM effects separately, while the combined impact on physiological and hormonal responses, particularly ABA signaling and stomatal regulation, remains poorly understood [2]. Understanding how AM fungi influence drought signaling in different cultivars is essential to optimizing cultivar selection and improving fruit resilience under climate stress. In this study, we evaluated two commercially important citrus cultivars, *Citrus macrophylla* Wester and *Citrus reshni* Hort. ex Tanaka (known as ‘Cleopatra’ mandarin). Both cultivars are widely used as rootstocks in Mediterranean orchards, and they are known to influence scion performance under abiotic stress. Several studies in semi-arid environments have documented the agronomic relevance of these cultivars in such contexts [5,9]. These features make Cleopatra and *C. macrophylla* a meaningful contrast for exploring how AM symbiosis interacts with cultivar-specific drought strategies in citrus [7,8,12]. We evaluated the physiological and hormonal responses of *C. macrophylla* and Cleopatra inoculated with AM fungi and subjected to progressive soil drying. Our objectives were to (i) assess how AM symbiosis affects gas exchange, stomatal conductance, and ABA accumulation in roots and leaves; (ii) compare the magnitude and direction of these responses between contrasting cultivars; and (iii) discuss the implications of these cultivar-specific responses in improving plant resilience under water-limited and climate-change conditions. We hypothesized that AM fungi inoculation would enhance photosynthesis and water-use efficiency as soil moisture decreases and that the main mechanisms behind this improvement—hydraulic support versus ABA-driven signaling—would vary between cultivars, leading to distinct patterns in stomatal behavior, *A* − *g_s_* relationships, and ABA distribution between roots and leaves. This work contributes to a better understanding of cultivar–mycorrhiza relationships and their potential application in sustainable citrus production in increasingly arid environments.

## 2. Results

### 2.1. Mycorrhizal Colonization and Initial Plant Status

**Figure 1:**
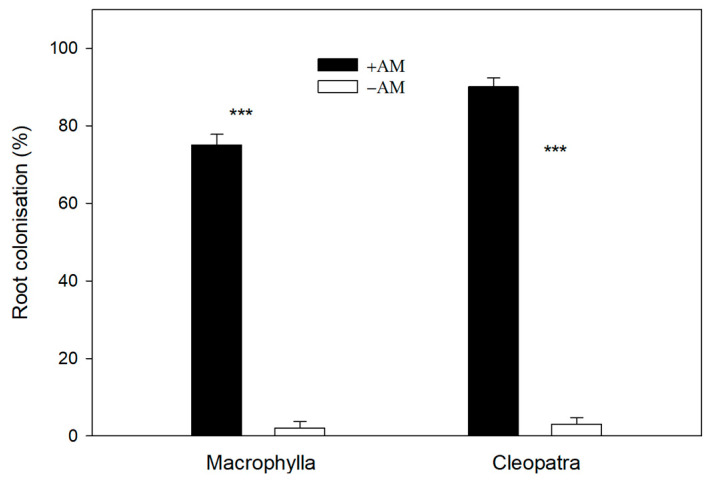
Root colonization on mycorrhizal (+AM) and non-mycorrhizal (−AM) plants for both Macrophylla and Cleopatra cultivars before starting the drying process. *** indicates significant at *p* < 0.001.

Microscopic analysis confirmed successful colonization in +AM plants of both cultivars, whereas −AM plants showed negligible colonization (Figure 1). The level of colonization differed slightly between cultivars, with Cleopatra exhibiting more fungal structures than Macrophylla, although both showed consistent and effective AM establishment.

At the onset of the experiment, the two-way ANOVA detected a cultivar effect across all growth traits and an AM main effect on leaf area, root fresh weight, and root/shoot ratio, with no cultivar × AM interaction (Table 1). Plant fresh weight, shoot fresh weight, and plant height did not show an AM effect (Table 1).

Leaf mineral concentrations also showed main effects of the cultivar and of AM on N, P, and Ca, without a cultivar × AM interaction (Table 2). The remaining elements did not exhibit a consistent AM main effect. Despite these statistically significant differences, the overall nutritional profiles remained within normal physiological ranges.

### 2.2. Gas Exchange and Water Status Under Varying Soil Moisture Conditions

**Figure 2:**
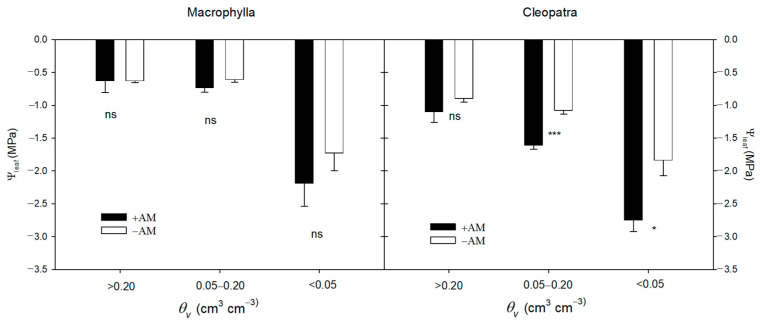
Leaf water potential (Ψ_leaf_) of +AM and −AM plants in three volumetric soil water content (*θ_v_*) ranges (>0.20, 0.05–0.20, <0.05 cm^3^ cm^−3^) for Macrophylla and Cleopatra cultivars. ‘ns’, *, and *** indicate not significant and significant at *p* < 0.05, and *p *< 0.001, respectively.

Under well-watered conditions (*θ_v_* > 0.20 cm^3^ cm^−3^), AM inoculation increased stomatal conductance (*g_s_*), photosynthetic rates (*A*), transpiration rates (*E*), and intrinsic water use efficiency (*A*/*g_s_*) in Macrophylla, whereas in Cleopatra, it increased *A* and *E* but not *g_s_* or *A*/*g_s_* (Table 3). Despite the higher transpiration in +AM plants, their leaf water potential (Ψ_leaf_) remained comparable to that of −AM plants under high soil moisture conditions (Figure 2).

**Figure 3:**
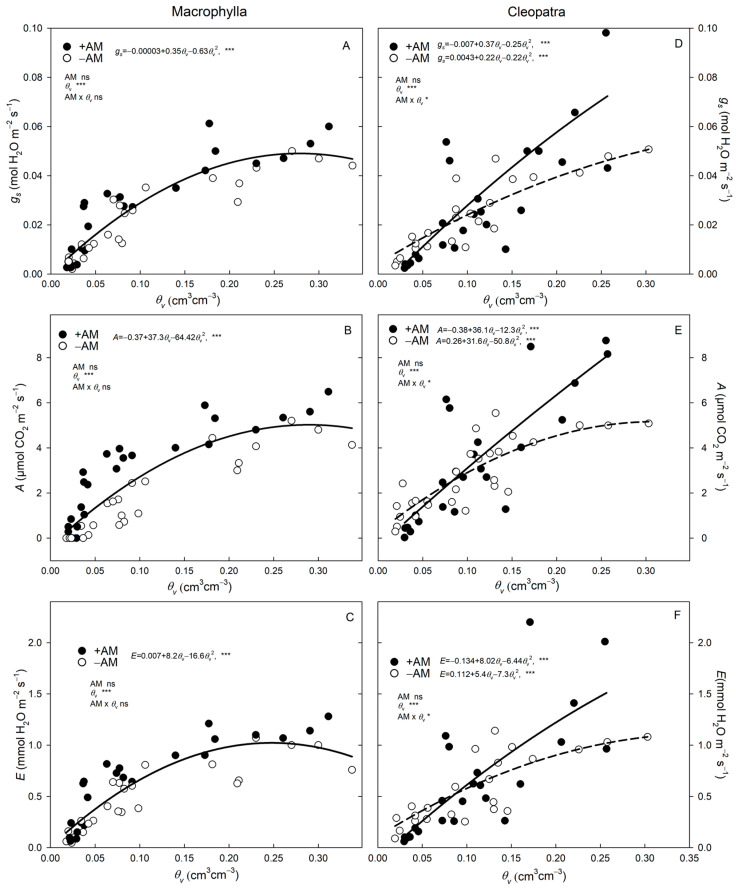
Gas-exchange responses to volumetric soil water content (*θ_v_*) in +AM and −AM plants. Lines are ANCOVA fits (AM + *θ_v_* + AM × *θ_v_*) within each cultivar. (**A**) Macrophylla: *A* vs. *θ_v_*. (**B**) Macrophylla: *g_s_* vs. *θ_v_*. (**C**) Macrophylla: *E* vs. *θ_v_*. (**D**) Cleopatra: *A* vs. *θ_v_*. (**E**) Cleopatra: *g_s_* vs. *θ_v_*. (**F**) Cleopatra: *E* vs. *θ_v_*. When the AM × covariate interaction is significant, solid lines represent +AM, and dashed lines represent −AM. When the interaction is non-significant, a single solid line shows the pooled ANCOVA slope for +AM and −AM combined; symbols differentiate treatments in the scatter plot. Dots are individual plants. ‘ns’, *, and *** indicate not significant and significant at *p* < 0.05, and *p *< 0.001, respectively.

During soil drying, the gas-exchange responses of Macrophylla and Cleopatra differed between +AM and −AM plants (Figure 3). Macrophylla plants reduced stomatal opening with decreasing soil moisture, but this response was gradual and similar in both +AM and −AM plants. In contrast, Cleopatra +AM plants closed their stomata faster than −AM plants as soil moisture decreased. When *θ_v_* ranged between 0.05 and 0.20 cm^3^ cm^−3^, +AM Macrophylla plants showed a marked rise in *A* (4.2 vs. 1.8 µmol CO_2_ m^−2^ s^−1^) with only a moderate increase in *g_s_* (0.038 vs. 0.024 mol H_2_O m^−2^ s^−1^), resulting in a significant gain in *A*/*g_s_* (115 vs. 76). In contrast, +AM Cleopatra plants displayed no significant changes in *A* (3.2 vs. 3.0) or *g_s_* (0.025 vs. 0.027), and consequently *A*/*g_s_* remained similar (131 vs. 119). These gas-exchange patterns explain why AM improves *A*/*g_s_* in Macrophylla under moderate drought conditions but not in Cleopatra. This advantageous behavior of +AM Macrophylla plants persisted even under very low soil moisture conditions (*θ_v_* < 0.05 cm^3^ cm^−3^). At these extreme soil moisture levels, stomatal conductance was very low and similar in both +AM and −AM Macrophylla plants, leading to reduced gas exchange in both groups. Nevertheless, +AM Macrophylla plants maintained a higher water use efficiency (*A*/*g_s_* and *A*/*E*) than −AM plants due to their higher *A* values (Table 3). In contrast, Cleopatra plants responded differently under these extreme conditions (Figure 3D–F), with a more pronounced stomatal closure in +AM plants than in −AM plants, resulting in reduced *E* and *A* in the inoculated group (Table 3).

The relationships between *A* and *E* with *g_s_*, revealed contrasting patterns between cultivars and AM treatments. The range of *g_s_* was markedly narrower in Macrophylla than in Cleopatra, especially in +AM plants. While −AM plants of both cultivars reached similar maximum *g_s_* values (0.045 and 0.050 mol H_2_O m^−2^ s^−1^, in Macrophylla and Cleopatra, respectively), +AM plants showed a contrasting behavior: Macrophylla peaked at 0.06 mol H_2_O m^−2^ s^−1^, whereas Cleopatra doubled this value (0.12 mol H_2_O m^−2^ s^−1^). Consequently, the range of *g_s_*, *A*, and *E* was greater in +AM plants than in −AM plants, and this effect was particularly pronounced in Cleopatra.

**Figure 4:**
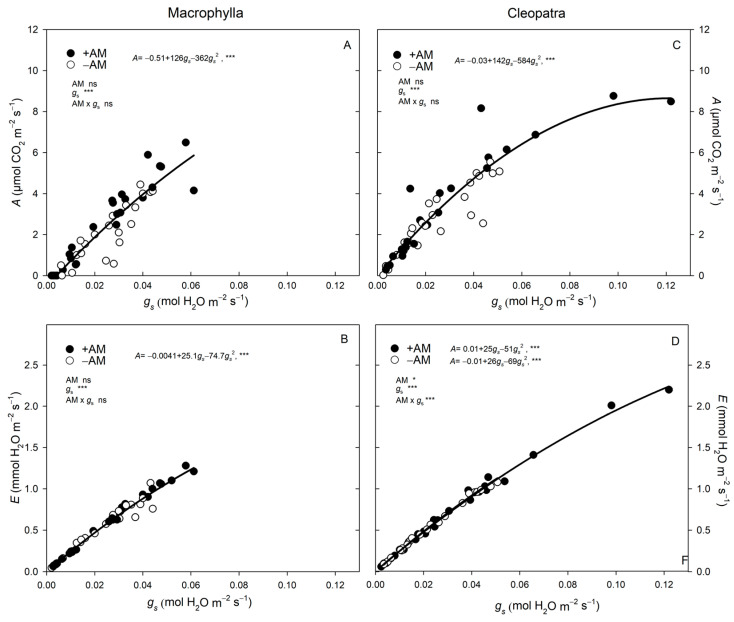
Relationships between *A* and *E* with *g_s_* in +AM and −AM plants, analyzed within each cultivar by an ANCOVA. Panels: (**A**) Macrophylla: *A* vs. *g_s_*. (**B**) Macrophylla: *E* vs. *g_s_*; (**C**) Cleopatra: *A* vs. *g_s_*; (**D**) Cleopatra: *E* vs. *g_s_*. Lines are ANCOVA fits (model: AM + *g_s_* + AM × *g_s_*). When the AM × covariate interaction is significant, solid lines represent +AM, and dashed lines represent −AM. When the interaction is non-significant, a single solid line shows the pooled ANCOVA slope for +AM and −AM combined; symbols differentiate treatments in the scatter plot. Dots are individual plants (independent sampling). The range of *g_s_* differs between cultivars because it reflects their intrinsic physiological behavior under the same experimental conditions (Cleopatra reached higher *g_s_* values than Macrophylla, which maintained a narrower range even under well-watered conditions. Panel 4D: both regression lines are plotted; due to overlap across the observed *g_s_* range, the AM− fit (dashed) is indistinguishable. ‘ns’, *, and *** indicate not significant and significant at *p* < 0.05, and *p *< 0.001, respectively.

In Cleopatra, the *A*–*g_s_* relationship followed a quadratic trend, with *A* approaching a plateau near *g_s_* ≈ 0.10 mol H_2_O m^−2^ s^−1^, while the *E*–*g_s_* curve continued to rise within the observed *g_s_* range. Beyond this *g_s_* threshold, further increases in *g_s_* did not enhance *A* but increased *E.* This suggests a differential coordination of photosynthesis and transpiration under high stomatal conductance conditions in Cleopatra cultivars. By contrast, in Macrophylla, *A* increased proportionally with *g_s_* across its narrower operational *g_s_* range, and no plateau was detected within the observed data (Figure 4A).

### 2.3. ABA Dynamics in Roots and Leaves in Response to Soil Drying

**Figure 5:**
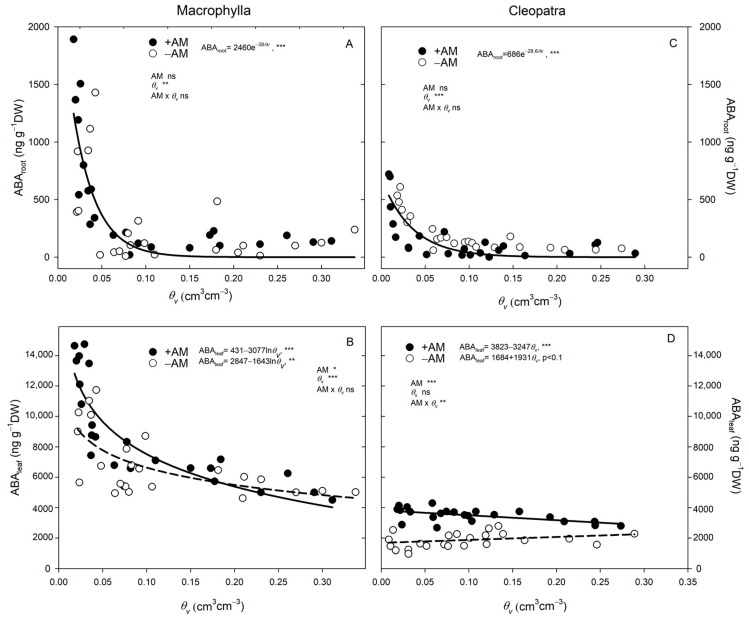
Abscisic acid concentrations in roots (ABA_root_) and leaves (ABA_leaf_) as a function of volumetric soil water content (*θ_v_*) in +AM and −AM plants, analyzed within each cultivar by an ANCOVA. (**A**) Macrophylla: ABA_root_ vs. *θ_v_*; (**B**) Macrophylla: ABA_leaf_ vs. *θ_v_*; (**C**) Cleopatra: ABA_root_ vs. *θ_v_*; (**D**) Cleopatra: ABA_leaf_ vs. *θ_v_*. Lines are ANCOVA fits (model: AM + *θ_v_* + AM × *θ_v_*) within each cultivar and tissue. When the AM × covariate interaction is significant, solid lines represent +AM, and dashed lines represent −AM. When the interaction is non-significant, a single solid line shows the pooled ANCOVA slope for +AM and −AM combined; symbols differentiate treatments in the scatter plot. Dots are individual plants. ‘ns’, *, **, and *** indicate not significant and significant at *p* < 0.05, *p* < 0.01, and *p *< 0.001, respectively.

ABA concentrations in Macrophylla plants were much higher than those found in Cleopatra plants, regardless of soil moisture levels or inoculation treatment (Table 4; Figure 5). In Macrophylla plants, ABA concentrations in roots (ABA_root_) and leaves (ABA_leaf_) were similar between +AM and −AM plants across the studied soil moisture categories, except under extreme drought conditions (*θ_v_* < 0.05 cm^3^ cm^−3^; Table 4). When soil moisture dropped below 0.05 cm^3^ cm^−3^, both +AM and −AM Macrophylla plants showed increased ABA_root_ and ABA_leaf_ concentrations as compared to those observed under higher soil moisture conditions. Under these drought conditions, ABA_leaf_ was significantly higher in +AM than in −AM plants (Table 4). Conversely, Cleopatra plants exhibited a completely different pattern: +AM plants consistently showed higher ABA_leaf_ concentrations than those of −AM plants at any given soil moisture level (Figure 5D; Table 4). Moreover, unlike the pattern observed in Macrophylla leaves, during the drying process, ABA_leaf_ in +AM plants increased only slightly as compared to levels under ideal soil moisture conditions, and no exponential increase was observed when soil moisture dropped below 0.05 cm^3^ cm^−3^ (Figure 5D). In contrast, ABA_root_ concentrations in Cleopatra plants increased sharply under severe drought conditions in both +AM and −AM plants, with +AM roots showing significantly higher ABA_root_ levels during moderate soil drying (*θ_v_* between 0.05 and 0.20 cm^3^ cm^−3^), indicating enhanced hormonal signaling from roots (Table 4).

### 2.4. Physiological Correlations

**Figure 6:**
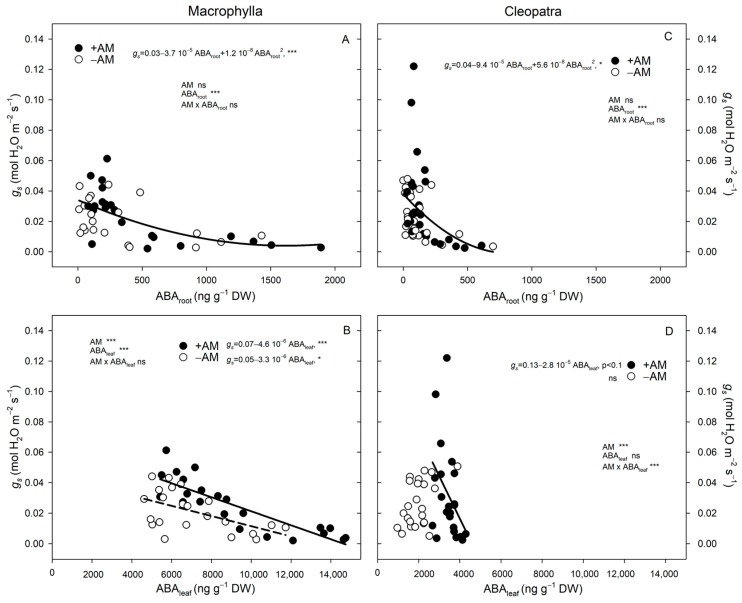
Stomatal conductance (*g_s_*) as a function of ABA in roots and leaves in +AM and −AM plants, analyzed within each cultivar by ANCOVA. (**A**) Macrophylla: *g_s_* vs. ABA_root_; (**B**) Macrophylla: *g_s_* vs. ABA_leaf_; (**C**) Cleopatra: *g_s_* vs. ABA_root_; (**D**) Cleopatra: *g_s_* vs. ABA_leaf_. Lines are ANCOVA fits (model: AM + ABA + AM × ABA) within each cultivar. When the AM × covariate interaction is significant, solid lines represent +AM, and dashed lines represent −AM. When the interaction is non-significant, a single solid line shows the pooled ANCOVA slope for +AM and −AM combined; symbols differentiate treatments in the scatter plot. Dots are individual plants. ‘ns’, *, and *** indicate not significant and significant at *p* < 0.05, and *p *< 0.001, respectively.

To explore the hormonal control underlying stomatal behavior in both Macrophylla and Cleopatra plants, the relationships between ABA concentrations in roots and leaves and stomatal conductance were analyzed (Figure 6). In both cultivars, a clear negative correlation was observed between ABA concentration and stomatal conductance, although the magnitude and pattern of these relationships differed markedly between Macrophylla and Cleopatra.

When both cultivars were compared, different patterns were observed. +AM Macrophylla plants maintained stomatal opening at a relatively high ABA_root_ concentration (Figure 6A); in Cleopatra, +AM plants tended to close stomata earlier, a response that was more closely associated with increases in ABA_root_ than with ABA_leaf_ (Figure 6C,D).

In Macrophylla, *g_s_* decreased as ABA_root_ increased, showing a clear negative relationship in both treatments (Figure 6A). The interaction between AM inoculation and ABA_root_ was not significant, indicating that the effect of ABA_root_ on *g_s_* was similar in +AM and −AM plants. For ABA_leaf_, *g_s_* also decreased with increasing ABA, and the two regression lines of +AM and −AM were nearly parallel, reflecting comparable slopes (Figure 6B). However, AM inoculation had a significant effect on the covariate analysis, meaning that for a given ABA_leaf_ concentration, +AM plants maintained a higher *g_s_* than −AM plants.

In contrast, Cleopatra displayed a different pattern. The ABA_root_–*g_s_* relationship was stronger than that of ABA_leaf_–*g_s_*, indicating that root-derived ABA exerted a greater control over stomatal closure in this cultivar (Figure 6). In +AM Cleopatra plants, increases in ABA_root_ coincided with reductions in *g_s_*, whereas ABA_leaf_ showed little change with soil drying.

## 3. Discussion

### 3.1. Starting Conditions and Mycorrhizal Colonization

Mycorrhizal colonization was observed only in +AM plants, while −AM plants showed a negligible infection (Figure 1), consistent with the strong mycotrophy reported in citrus [19,20,21,22]. The plant growth data showed that at the pre-drought baseline, +AM and −AM plants entered the drying period without dependence on the cultivar. On the other hand, the observed increase in +AM leaves in macronutrients such as N and P (Table 2) is consistent with published citrus leaf sufficiency ranges [23,24] and agrees with studies demonstrating that *R. irregularis* and *Funneliformis* species readily colonize citrus roots under controlled conditions and enhance the uptake of P and other nutrients [19,20,22]. These improvements, widely documented in citrus seedlings, are considered characteristic responses of early symbiosis under moderate or low nutrient availability conditions [19,22,25,26]. Variability in colonization and nutrient responses among cultivars has been linked to differences in root system architecture and AMF compatibility, which modulate nutrient uptake efficiency and the effectiveness of the mycorrhizal pathway [15].

### 3.2. Mycorrhiza-Driven Modulation of Gas Exchange and Water-Use Efficiency

Macrophylla and Cleopatra differed in stomatal behavior. Macrophylla operated over a narrow *g_s_* range with an approximately linear *A–g_s_* relation, whereas Cleopatra exhibited a wider *g_s_* range and showed a saturating *A–g_s_* response (Figure 4). These intrinsic differences helped explain the distinct gas-exchange responses to AM inoculation: in −AM plants; both cultivars reached a similar maximum *g_s_*, but in +AM plants Cleopatra peaked at roughly twice the *g_s_* of Macrophylla (Figure 4). Consequently, +AM plants (especially Cleopatra) exhibited wider operational ranges of *g_s_*, *A*, and *E*, indicating that mycorrhization expands the gas-exchange window, also revealing the greater stomatal capacity in Cleopatra. In +AM Cleopatra plants at high *g_s_*, *A* tended to plateau while *E* continued to rise, which decreased *A/E* and *A/g_s_*; thus, the mycorrhizal advantage was apparent mainly below that range (Figure 4C,D; Table 3). By contrast, Macrophylla showed an approximately linear *A–g_s_* relation over a narrower *g_s_* range, and +AM and −AM behaved similarly across that range (Figure 4), indicating a tight stomatal regulation consistent with a conservative response under soil drying conditions.

These cultivar-specific differences became clearer along the soil moisture gradient. In Macrophylla, AM inoculation enhanced performance across a narrow, tightly regulated stomatal range, and the near-linear *A*–*g_s_* relation was maintained across moisture levels (Figure 3 and Figure 4). The higher *A* in +AM under well-watered and moderate drought conditions occurred without an additional transpirational cost, consistent with hydraulic/metabolic support—improved root hydraulics and aquaporin regulation, osmotic adjustment, and greater nutrient uptake—rather than higher stomatal opening [2,13,16], in line with evidence in citrus and other species [14,15]. Although AM increased *A/g_s_* and *A/E* in both cultivars, the effect was statistically evident only in Macrophylla (Table 3). Under moderate (*θ_v_* = 0.05–0.20 cm^3^ cm^−3^) and severe drying (*θ_v_* < 0.05 cm^3^ cm^−3^) conditions, +AM mitigated the photosynthetic penalty of stomatal closure, indicating a partial relief of stomatal limitation. Related microbial symbioses can also enhance photosynthetic capacity under water deficit via adjustments in chlorophyll biosynthesis [27]. Consistent with work in trifoliate orange showing that AM can increase *A* and *g_s_* while lowering bioactive GA_3_ under drought conditions [28], Macrophylla exhibits a drought-avoidance pattern with tight stomatal regulation, and AM symbiosis confers a modest but functionally relevant gain in carbon assimilation with limited hydraulic risk.

In Cleopatra, +AM plants operated over a wider *g_s_* range, supporting a higher *A* under well-watered conditions (Figure 4C). As soil moisture decreased, *g_s_* decreased earlier, especially in +AM, indicating an opportunistic then conservative stomatal strategy (Figure 3D–F). This behavior was consistent with an isohydric tendency that prioritized hydraulic safety over carbon gain [1,3] and aligned with the enhanced ABA_root_ signal in this cultivar (Figure 5 and Figure 6).

These results align with meta-analyses showing that, under drought, AM fungi most consistently increase the photosynthetic rate among gas-exchange traits [13,29] and with reports that AM fungi improve drought tolerance and photosynthetic performance in citrus, with host-dependent effect sizes [15,30]. The saturating *A–g_s_* pattern in Cleopatra +AM is also consistent with AM-driven changes in stomatal sensitivity and carbon gain under fluctuating water availability conditions [2,14] and with observations in citrus under comparable conditions [31,32].

Our data support distinct AM-driven controls in each cultivar. In Macrophylla, AM plants maintained a higher *A* at comparable or lower *g_s_* under moderate drying conditions (Table 3), consistent with hydraulic/metabolic support—enhanced membrane transport and aquaporin regulation, osmotic adjustment, and antioxidant capacity—that delays stomatal limitation [13,29,31,32]. In Cleopatra, AM amplified ABA_root_ signaling, promoting earlier conservative stomatal control [14,33]. Both routes can improve WUE but through different physiological pathways, reinforcing cultivar-specific AM management under water limitation conditions. In Macrophylla, the narrow *g_s_* range and near-linear *A–g_s_* relation, together with stable gas exchange and a delayed ABA response, are consistent with a regulated, mildly anisohydric tendency that maintains carbon gain despite moderate declines in leaf water potential [1,3].

### 3.3. Hormonal Regulation and Cultivar-Specific Signaling

During progressive soil drying, ABA profiles indicated a cultivar-dependent modulation of ABA signaling by AM symbiosis. In both cultivars, ABA_root_ and ABA_leaf_ increased as *θ_v_* decreased (Figure 5; Table 4), but the magnitude, dynamics, and likely source of the signal differed between Macrophylla and Cleopatra.

In Macrophylla, ABA_root_ and ABA_leaf_ rose only slightly at moderate drying (*θ_v_* = 0.05–0.20 cm^3^ cm^−3^) and increased sharply only under severe drought conditions (*θ_v_* < 0.05 cm^3^ cm^−3^), when ABA_leaf_ was higher in +AM than −AM (Table 4). Thus, ABA_leaf_ accumulation was a late response. At moderate drying, despite comparable ABA_root_ concentrations between treatments, the early performance advantage in +AM Macrophylla could not be attributed to ABA. Beyond ABA, several AM-linked, non-hormonal processes documented in citrus likely supported the +AM Macrophylla response: (i) enhanced membrane transport and water relations (higher PM H^+^-ATPase activity and context-dependent aquaporin regulation), stabilizing leaf water status under soil drying conditions [13,32]; (ii) osmotic adjustment (higher soluble sugars and K^+^/Ca^2+^ in leaves and roots) [31]; and (iii) strengthened antioxidant capacity and membrane stability via increased root fatty-acid unsaturation, lowering ROS and lipid peroxidation [16,34,35]. Together, these mechanisms can partly decouple stomatal behavior from increasing ABA concentrations during early drought, allowing +AM Macrophylla to sustain gas exchange for a longer period of time.

In Cleopatra, +AM plants showed consistently higher ABA_leaf_ than −AM across all *θ_v_* classes, but ABA_leaf_ changed little with drying (Table 4; Figure 5D). By contrast, ABA_root_ increased sharply as *θ_v_* decreased and was higher in +AM, especially during moderate and severe drying (Table 4). This pattern supported a predominantly root-sourced signal driving earlier stomatal closure, whereas ABA_leaf_ likely reflected transport/compartmentation/turnover, consistent with root-to-shoot ABA translocation [33] and AM-amplified root-sourced signaling [14] Accordingly, ABA_root_, not the relatively stable ABA_leaf_, correlated more closely with the decline in *g_s_* in +AM Cleopatra as soil moisture decreased (Figure 6).

Our results support two AM-mediated routes. In Macrophylla, AM maintained stomatal opening and photosynthesis during moderate drying, without early increases in ABA_root_ or ABA_leaf_; improvements in water acquisition, osmotic adjustment, soil/root hydraulic function, and antioxidant protection likely reduced reliance on ABA-mediated stomatal control, consistent with other AMF systems [36]. In Cleopatra, AM increased ABA_root_ concentrations, promoting earlier stomatal closure and a more conservative, hydraulically safer water-use strategy [2]. In summary, AM symbiosis modulates ABA signaling in a cultivar-dependent manner. In Cleopatra, earlier stomatal closure in +AM was linked to enhanced ABA_root_; in Macrophylla, sustained gas exchange under moderate stress was better explained by AM-driven hydraulic/metabolic stabilization than by hormonal changes. These contrasts highlight cultivar-specific AM–ABA interactions, consistent with reports in citrus and other species [2,14] and with meta-analytic evidence that AM fungi effects on *g_s_* and *A* vary widely across hosts and conditions [13].

### 3.4. Practical Implications and Future Perspectives

AM effects are strongly cultivar-dependent. AM inoculation should be applied with cultivar-specific criteria rather than as a general practice. From a management perspective, +AM Macrophylla sustained higher *A* under moderate drought conditions, whereas +AM Cleopatra showed an earlier stomatal closure; accordingly, AM can support irrigation-saving regimes such as regulated deficit irrigation (RDI) or partial rootzone drying (PRD). Operationally, Macrophylla’s sustained *A* at low–moderate soil water supports RDI (water saving with stable yield, higher WUE) [37,38], while Cleopatra’s earlier closure suits alternating PRD, limiting transpiration and improving irrigation WUE [7,11,37]. Field applications should also protect AM establishment, avoiding excess soil moisture and high phosphorus inputs [39].

Future work should prioritize (i) field validation of the cultivar-specific responses; (ii) refinement of inoculation protocols with longitudinal monitoring of AM establishment; and (iii) identification of cultivar traits that enhance AM effectiveness using physiological and phenotyping tools [40]. These steps will enable evidence-based, cultivar-specific inoculation.

In summary, AM fungi can improve drought tolerance and water-use efficiency in citrus when strategies are tailored to cultivar physiology and aligned with orchard soil and irrigation conditions.

## 4. Materials and Methods

### 4.1. Plant Culture and Experimental Design

The experiment was carried out in a walk-in controlled environment room (3 × 6.5 m) at the Instituto Murciano de Investigación y Desarrollo Agrario y Medioambiental (IMIDA), La Alberca, Murcia, Spain, from March 2022 to July 2022. The photoperiod was 16 h (07.00–23.00 h); air temperature varied within 20–24 °C and relative humidity within 65–85% in the same room. Seeds of *Cleopatra mandarin* and *Citrus macrophylla* were surface sterilized for 10 min in 20% NaClO, rinsed four times with sterile distilled water, and germinated into plastic trays containing moistened vermiculite. Forty-day-old seedlings were transplanted into plastic pots (1.1 L) containing substrate. The substrate was a mixture of silica filtration sand (particle size 0.4–0.8 mm) and clay-loam soil (soil/sand 1:3, *v/v*) that was sterilized in an autoclave for 1 h at 100 °C three times on alternate days.

Twenty-five grams of *Rhizophagus irregularis* (Blaszk., Wubet, Renker & Buscot) C. Walker & A. Schübler and *Funneliformis mosseae* (T.H. Nicolson & Gerd.) C. Walker & A. Schübler in equal shares (a mix of spores, mycorrhized roots, and substrate, with a density of ~1200 propagules g^−1^) and mycorrhizal inocula, propagated with the hybrid of *Sorghum bicolor* (L.) Moench and *Sorghum sudanense* (Piper) Hitch (*Sorghum bicolor* × *sudanense*) as a trap plant, were inoculated into the pots before transplanting. The inoculum was supplied by the Mycology-Mycorrhizas-Plant Biotechnology Laboratory, Department of Plant Biology, University of Murcia (Spain). Plants were irrigated with modified Hoagland’s solution [41] composed of 6 mM KNO_3_, 4 mM Ca(NO_3_)_2_·4H_2_O, 2 mM NH_4_H_2_PO_4_, 1 mM MgSO_4_·7H_2_O, 42.7 μM EDDHAFe, 25 μM H_3_BO_3_, 2 μM MnSO_4_, 2 μM ZnSO_4_, 0.5 μM CuSO_4_, and 0.065 μM (NH_4_)_6_Mo_7_O_24_. The non-inoculated plants (−AM) were watered with this solution with 2 mM H_2_PO_4_^–^, and inoculated plants (+AM) were irrigated with the same solution, but P. Phosphorus was omitted from the nutrient solution in +AM plants to avoid the well-known suppression of arbuscular mycorrhization by elevated phosphate [42]. An adequate P supply to +AM plants was ensured by the growth substrate (soil/sand 1:3, Olsen P = 70 ppm, very high), from which AM fungi can mobilize P [22]. All plants were watered every other day with 250 mL of nutrient solution, a sufficient volume to maintain soil moisture near field capacity, and to produce leachate from the bottom of all pots, thus avoiding any build-up of salts. The plants were randomly placed in the same growth chamber. To prevent cross-contamination, +AM and −AM plants were maintained in separate groups that were never intermixed. Within each group, 60 pots (30 per cultivar) were randomly interspersed in a 12 × 5 grid and repositioned weekly using a cyclic row rotation (moving the five pots at one end to the opposite end and shifting all rows forward by one position) to reduce positional/edge effects.

Before drought imposition, 7 seedlings per cultivar × AM treatment were used for pre-drought baseline characterization. The remaining 23 seedlings per combination then entered the drying sequence: sixty days after planting, all remaining plants were watered to field capacity and subsequently subjected to a continuous soil drying episode by withholding water from pots. Pairs of +AM and −AM plants of each cultivar were randomly sampled every day from the beginning of the drying period until the complete drying of the soil. Pre-drought traits were evaluated on seven plants per treatment combination. During the drying episode (15 days), independent plants were harvested daily from each combination. On each sampling day, plants were paired within cultivar by treatment: days 1–7, one +AM and one −AM plant per cultivar; days 8–15, two +AM and two −AM plants per cultivar. No seedling mortality occurred throughout the experiment; therefore, no plants were replaced.

Gas-exchange and leaf water potential were measured immediately before harvest. Before starting the soil drying process, we verified the establishment of the AM symbiosis and assessed whether +AM and −AM plants differed in their initial growth or nutritional status. This characterization ensured that subsequent physiological and hormonal responses could be attributed to drought progression and AM inoculation rather than to pre-existing differences between treatments.

### 4.2. Physiological Determinations

Every day during the drying period, and before the plants were harvested, physiological measurements were made. Leaf gas-exchange parameters (photosynthetic rate *A*, transpiration rate *E*, stomatal conductance *g_s_*, intrinsic water use efficiency *A*/*g_s_*, and instantaneous water use efficiency *A*/*E*) were measured in a single youngest fully expanded leaf per plant using a portable photosynthesis system (Li-6400, Li-Cor, Lincoln, NE, USA) equipped with a broad leaf chamber (6.0 cm^2^). The air flow rate inside the leaf chamber was 300 μmol s^−1^, and the temperature of the block of the leaf chamber was fixed at 24 °C. Portable 12 g cartridges of high-pressure, liquefied, pure CO_2_ were attached to the console by an external CO_2_ source assembly and were controlled automatically by a CO_2_ injector system (6400-01 Li-Cor, Lincoln, NE, USA). The reference CO_2_ concentration was fixed at 450 μmol CO_2_ mol^−1^. All the measurements were made using a red-blue light source (6400-02B light-emitting diode; Li-Cor, Lincoln, NE, USA) attached to the leaf chamber, and the PPFD was fixed at 400 μmolm^−2^ s^−1^. Following the gas-exchange measurements, leaf water potential (Ψ_leaf_) was measured in the same leaf using a Schölander-type pressure chamber (model 3000; Soil Moisture Equipment. Corp., Goleta, CA, USA) [43].

### 4.3. Plant Growth and Mineral Analysis

Before harvesting, plant growth was estimated in each plant by counting the leaves and measuring the plant height. After that, the plants were defoliated, and plant leaf area was determined by a LI-3100 Area Meter (LI‑COR Biosciences, Lincoln, NE, USA). Roots were separated carefully from the soil and washed with distilled water. After root pieces were taken for estimation of ABA and mycorrhizal colonization, the root system and all tissue fractions from the shoot were fresh weighed and oven-dried at 60 °C until constant weight for dry weight determination.

Dried leaves were ground and stored for chemical analysis. The plant tissues (250 mg) were ashed at 550 °C, the ashes were dissolved in 0.7 N HNO_3_, and then P, K, Mg, Ca, Fe, Cu, Mn, and B were analyzed by an Inductively Coupled Plasma Optical Emission Spectrometer (Vista‑MPX; Varian Australia Pty Ltd., Mulgrave, VIC, Australia). Nitrogen concentration was determined by combustion [44] using a total N analyzer FP528 LC (LECO Truspec, St. Joseph, MI, USA).

### 4.4. ABA Determination

From every seedling, two similar leaves to those used for physiological determinations and 200 mg of fine fresh roots taken from the middle part of the root system were collected for ABA determination and stored in liquid nitrogen. Tissue samples were freeze-dried, ground, and extracted with deionized water at a 1:50 ratio. ABA concentrations in leaves and roots samples were analyzed by a radioimmunoassay [45] using a monoclonal antibody AFRC MAC 252.

### 4.5. Determination of Mycorrhizal Colonization

For every plant, root pieces were taken from the middle part of the root system for estimation of mycorrhizal colonization. Samples were cleaned and stained with trypan blue according to the method of Phillips and Haymann. Root samples were cleared in KOH and stained with trypan blue prepared in a lactic acid/glycerol/water solution (1:1:1, *v/v/v*), following the phenol-free modification of Phillips and Hayman [46]. We adopted the lactic acid formulation to avoid the toxicity and corrosivity associated with lactophenol, while maintaining excellent stain uptake of mycorrhizal structures. One hundred root segments per plant were mounted on slides, squashed by pressing on the coverslips, and quantified for AM colonization according to McGonigle et al. [47].

### 4.6. Soil Water Content

After the plants were removed, the soil water content of each pot of an individual plant was also determined by the gravimetric method. Soil samples from each pot were weighed (W_w_), dried at 65 °C to a constant weight, and re-weighed (W_d_). Gravimetric soil water was calculated according to Kasischke et al. [48] as W_g_ = (W_w_ − W_d_) × 100/W_d_. Volumetric soil water content (*θ_v_*) was calculated as *θ_v_* = W_g_ × ρ, where ρ was the soil bulk density, which was previously calculated.

### 4.7. Statistical Analysis

Analyses were performed per cultivar. Pre-drought traits (Table 1 and Table 2) were analyzed by a two-way ANOVA with cultivar and AM fixed factors, including the cultivar × AM interaction. Gas exchange within soil moisture categories defined by ranges (*θ_v_* > 0.20, 0.05–0.20, <0.05 cm^3^ cm^−3^) were tested by a one-way ANOVA with AM (+AM, −AM) as the fixed factor. Along continuous gradients, an ANCOVA was used with AM (fixed) and the relevant covariate: *θ_v_* (Figure 3), *g_s_* (Figure 4), ABA_root_, and ABA_leaf_ (Figure 5 and Figure 6); the AM × covariate interaction tested slope homogeneity. If AM × covariate was non-significant, the treatment comparisons used adjusted means as the covariate mean; if significant, the inference focused on treatment-specific slopes. In Cleopatra, the curvature in *A*–*g_s_* was evaluated with a quadratic term, and the model choice was guided by Schwarz’s Bayesian Criterion (SBC/BIC). Assumptions (linearity, normality, and homoscedasticity) were checked; log or Box–Cox transformations were applied when required. Tests were two-tailed with α = 0.05. The ANOVA/ANCOVA were run in Statgraphics Plus 2.0 and the regressions and SBC in SigmaPlot 11.0.

## 5. Conclusions

AM inoculation improved drought responses in both citrus cultivars but through different mechanisms that reflected their intrinsic physiological strategies. In Macrophylla, AM plants maintained higher stomatal conductance and photosynthesis than non-inoculated plants during moderate soil drying, despite showing similar ABA levels in roots and leaves. This indicates that the benefits of AM symbiosis in this cultivar are mainly associated with improved hydraulic and/or metabolic functioning, rather than early hormonal regulation. Only under severe drought conditions did AM Macrophylla plants show a clear increase in foliar ABA.

In Cleopatra, AM inoculation produced a different pattern: AM plants showed earlier stomatal closure and higher ABA concentrations in roots across the drying cycle. These data indicate that AM symbiosis strengthened root-derived ABA signaling, leading to a more conservative water-use strategy. Although AM Cleopatra plants reached a higher maximum *g_s_* and photosynthesis under well-watered conditions, this advantage disappeared once stomatal conductance exceeded a threshold at which photosynthesis no longer increased and water loss continued.

These results demonstrate that AM symbiosis does not generate a uniform response in citrus cultivars but instead interacts with the intrinsic physiological behavior of each cultivar. In Macrophylla, AM fungi helped sustain carbon assimilation under stress; in Cleopatra, they promoted an earlier stomatal control. These findings highlight that the effectiveness of AM inoculation strongly depends on the cultivar and underline the potential of AM fungi as a tool to support drought management in citrus when applied within cultivar-specific frameworks.

## Figures and Tables

**Table 1:** Plant growth at the start of the drying experiment in Macrophylla and Cleopatra, for +AM and −AM within each cultivar. Root/Shoot indicates the ratio of root fresh weight to shoot fresh weight.

Cultivar	AM	Plant Fresh Weight (g)	Root Fresh Weight (g)	Shoot Fresh Weight (g)	Root/ Shoot	Foliar Area (cm^2^)	Plant Height (cm)
Macrophylla	+AM	7.27 ± 0.23	3.12 ± 0.24	4.15 ± 0.14	0.760 ± 0.065	167.6 ± 5.7	24.2 ± 0.5
	−AM	7.10 ± 0.32	3.30 ± 0.19	3.80 ± 0.18	0.874 ± 0.049	150.7 ± 4.3	24.9 ± 0.5
Cleopatra	+AM	13.25 ± 0.44	4.26 ± 0.21	9.00 ± 0.32	0.475 ± 0.024	356.9 ± 12.6	42.5 ± 0.6
	−AM	13.06 ± 0.26	4.83 ± 0.21	8.23 ± 0.29	0.587 ± 0.024	321.5 ± 8.7	41.2 ± 0.3
Macrophylla		7.18 ± 0.19	3.21 ± 0.15	3.97 ± 0.12	0.817 ± 0.042	159.2 ± 4.1	24.5 ± 0.4
Cleopatra		13.17 ± 0.26	4.51 ± 0.17	8.66 ± 0.22	0.525 ± 0.025	341.2 ± 9.8	41.9 ± 0.4
	+AM	9.76 ± 0.92	3.59 ± 0.23	9.76 ± 0.92	0.641 ± 0.057	246.5 ± 28.7	31.8 ± 2.7
	−AM	9.27 ± 0.89	3.86 ± 0.26	9.27 ± 0.89	0.770 ± 0.051	212.8 ± 25.2	30.8 ± 2.4
		ANOVA					
Cultivar		***	***	***	***	***	***
AM		ns	*	ns	*	**	ns
Cultivar × AM		ns	ns	ns	ns	ns	ns

‘ns’, *, **, and *** indicate not significant and significant at *p* < 0.05, *p* < 0.01, and *p* < 0.001, respectively. Values are means ± SE; n = 7 per treatment combination (cultivar × AM). Marginal means by cultivar or AM are based on n = 14 (pooled across the other factor).

**Table 2:** Effect of mycorrhizal associations on leaf mineral nutrition of Macrophylla and Cleopatra plants at the beginning of the drying experiment for +AM and −AM within each cultivar. N, K, Ca, and Mg in %; Fe, Cu, Mn, Zn, and B in mg kg^−1^ DW.

Cultivar	AM	N	P	K	Ca	Mg	Fe	Cu	Mn	Zn	B
Macrophylla	+AM	2.61 ± 0.10	0.11 ± 0.01	1.95 ± 0.07	1.37 ± 0.08	0.24 ± 0.01	118 ± 8	25.6 ± 2.3	31.6 ± 3.0	20.7 ± 3.6	53.6 ± 2.0
	−AM	2.24 ± 0.04	0.09 ± 0.00	1.79 ± 0.09	1.50 ± 0.06	0.26 ± 0.01	99 ± 10	21.7 ± 2.3	40.2 ± 4.6	22.6 ± 4.4	52.2 ± 2.4
Cleopatra	+AM	2.56 ± 0.07	0.15 ± 0.00	1.50 ± 0.06	1.97 ± 0.08	0.30 ± 0.01	50 ± 3	11.4 ± 0.6	10.6 ± 0.7	21.4 ± 0.8	31.4 ± 1.5
	−AM	2.26 ± 0.03	0.10 ± 0.00	1.47 ± 0.05	2.22 ± 0.04	0.28 ± 0.01	42 ± 2	9.2 ± 0.5	13.9 ± 1.3	14.7 ± 0.3	36.6 ± 0.8
Macrophylla		2.43 ± 0.07	0.10 ± 0.00	1.87 ± 0.06	1.44 ± 0.05	0.25 ± 0.01	108 ± 7	23.7 ± 1.6	35.9 ± 2.9	21.7 ± 2.8	52.9 ± 1.5
Cleopatra		2.41 ± 0.06	0.12 ± 0.01	1.48 ± 0.04	2.09 ± 0.06	0.29 ± 0.01	46 ± 2	10.3 ± 0.5	12.3 ± 0.8	28.0 ± 1.1	34.0 ± 1.1
	+AM	2.59 ± 0.06	0.13 ± 0.01	1.74 ± 0.08	1.64 ± 0.10	0.27 ± 0.01	87 ± 11	19.1 ± 2.4	21.9 ± 3.4	21.0 ± 1.9	43.4 ± 3.4
	−AM	2.25 ± 0.02	0.10 ± 0.00	1.64 ± 0.07	1.83 ± 0.11	0.27 ± 0.01	73 ± 10	15.9 ± 2.2	28.0 ± 4.5	19.0 ± 2.6	45.0 ± 2.6
		ANOVA									
Cultivar		ns	*	***	***	***	***	***	***	ns	***
AM		***	***	ns	**	ns	ns	ns	ns	ns	ns
Cultivar × AM		ns	ns	ns	ns	ns	ns	ns	ns	ns	ns

‘ns’, *, **, and *** indicate not significant and significant at *p* < 0.05, *p* < 0.01, and *p* < 0.001, respectively. Values are means ± SE; n = 7 per treatment combination (cultivar × AM). Marginal means by cultivar or AM are based on n = 14 (pooled across the other factor).

**Table 3:** Leaf gas exchange for +AM and −AM plants in three volumetric soil water content (*θ_v_*) ranges for Macrophylla and Cleopatra cultivars. *g_s_* stomatal conductance (mol H_2_O m^−2^ s^−1^); *A*: CO_2_ assimilation rate (µmol CO_2_ m^−2^ s^−1^); *E*: transpiration rate (mmol H_2_O m^−2^ s^−1^); *A/g_s_* intrinsic water use efficiency (µmol CO_2_ mol^−1^ H_2_O); *A/E*: instantaneous water use efficiency (µmol CO_2_ mmol^−1^ H_2_O).

*θ_v_* (cm^3^ cm^−3^)	AM	Macrophylla					Cleopatra				
		*g_s_*	*A*	*E*	*A/g_s_*	*A/E*	*g_s_*	*A*	*E*	*A/g_s_*	*A/E*
>0.20	+AM	0.052 ± 0.005	5.9 ± 0.6	1.17 ± 0.11	113 ± 3	5.0 ± 0.4	0.069 ± 0.016	7.9 ± 0.6	1.46 ± 0.30	128 ± 27	5.9 ± 1.3
	−AM	0.038 ± 0.003	3.6 ± 0.3	0.78 ± 0.10	95 ± 1	4.8 ± 0.0	0.047 ± 0.003	5.0 ± 0.0	1.02 ± 0.04	109 ± 10	4.9 ± 0.2
ANOVA		*	**	*	**	ns	ns	*	ns	ns	ns
0.05–0.20	+AM	0.038 ± 0.004	4.2 ± 0.4	0.85 ± 0.07	115 ± 11	5.0 ± 0.5	0.025 ± 0.004	3.2 ± 0.5	0.57 ± 0.08	131 ± 5	5.4 ± 0.2
	−AM	0.024 ± 0.003	1.8 ± 0.3	0.56 ± 0.06	76 ± 8	3.2 ± 0.3	0.027 ± 0.003	3.0 ± 0.3	0.63 ± 0.006	119 ± 7	5.0 ± 0.3
ANOVA		***	*	**	**	*	ns	ns	ns	ns	ns
<0.05	+AM	0.011 ± 0.003	1.03 ± 0.34	0.26 ± 0.07	97 ± 9	4.2 ± 0.4	0.005 ± 0.001	0.49 ± 0.14	0.12 ± 0.02	103 ± 8	3.8 ± 0.3
	−AM	0.007 ± 0.02	0.18 ± 0.10	0.16 ± 0.03	34 ± 7	1.6 ± 0.3	0.011 ± 0.002	1.28 ± 0.24	0.28 ± 0.05	113 ± 11	4.6 ± 0.8
ANOVA		ns	*	ns	*	*	*	*	*	ns	ns

‘ns’, *, **, and *** indicate not significant and significant at *p* < 0.05, *p* < 0.01, and *p* < 0.001, respectively.

**Table 4:** ABA concentration in roots and leaves (ng g^−1^ DW) for +AM and −AM plants across *θ_v_* ranges in Macrophylla and Cleopatra.

*θ_v_* (cm^3^ cm^−3^)	AM	Macrophylla		Cleopatra	
		ABA_root_ (ng g^−1^ DW)	ABA_leaf_ (ng g^−1^ DW)	ABA_root_ (ng g^−1^ DW)	ABA_leaf_ (ng g^−1^ DW)
>0.20	+AM	144 ± 27	6711 ± 336	78 ± 11	2945 ± 109
	−AM	209 ± 57	5595 ± 303	63 ± 6	1934 ± 79
ANOVA		ns	ns	ns	**
0.05–0.20	+AM	165 ± 51	6406 ± 436	131 ± 13	3536 ± 99
	−AM	117 ± 31	6347 ± 232	45 ± 12	1982 ± 61
ANOVA		ns	ns	*	***
<0.05	+AM	845 ± 182	11,603 ± 865	449 ± 81	3921 ± 165
	−AM	656 ± 168	8758 ± 811	279 ± 57	1643 ± 73
ANOVA		ns	*	ns	***

‘ns’, *, **, and *** indicate not significant and significant at *p* < 0.05, *p* < 0.01, and *p* < 0.001, respectively.

## Data Availability

Data is contained within the article.

## Abbreviations

The following abbreviations are used in this manuscript:

*A*	Photosynthetic rate
*A/E*	Instantaneous water use efficiency
*A/g_s_*	Intrinsic water use efficiency
ABA	Abscisic acid
AM	Arbuscular mycorrhizal
*E*	Transpiration rate
*g_s_*	Stomatal conductance
ROS	Reactive oxygen species
*θ_v_*	Volumetric soil water content
Ψ_leaf_	Leaf water potential
